# Complete mitochondrial genome of *cylicocyclus auriculatus*: molecular structure and phylogenetic analysis

**DOI:** 10.1080/23802359.2022.2044928

**Published:** 2022-02-24

**Authors:** Xuankai Li, Lidan Wang, Yijun Chen, Xianxiang Wang, Xuan Zhou, Yue Xie

**Affiliations:** a Department of Parasitology, College of Veterinary Medicine, Sichuan Agricultural University, Chengdu, China; b College of Science, Sichuan Agricultural University, Ya'an, China

**Keywords:** Bursate nematode, *Cylicocyclus*, mtDNA, phylogenetic relationships

## Abstract

*Cylicocyclus* spp. (Nematoda: Strongylida: Cyathostominae) are the common and important parasitic nematodes found in horses and donkeys worldwide. In this study, the complete mitochondrial genome of *Cylicocyclus auriculatus* Looss 1900, a representative member of this genus from the donkey in Southwest China was determined using the next-generation DNA sequencing technology. The genome was 13,851 bp in size and consisted of 36 genes including 12 protein-coding genes (*atp*6, *cox*1-3, *cytb*, *nad*1-6 and *nad*4L), 22 transfer RNA genes and two ribosomal RNA genes (*rrn*L and *rrn*S), as well as two non-coding regions. Phylogenetic analysis showed that *C. auriculatus* and *Cylicocyclus insigne* Boulenger 1917 were closely related, and then both grouped with other congeneric species and formed a monophyletic relationship with either species of *Cyathostomum*, *Coronocyclus*, *Cyathostomum*, *Cylicostephanus* or *Cylicodontophorus*, demonstrating their phylogenetic stability within Cyathostominae. These cumulative mitochondrial DNA data provide novel and useful genetic markers for molecular diagnostic, systematic and evolutionary biological studies of Cyathostominae nematodes.

*Cylicocyclus* parasites (Nematoda: Strongylida: Cyathostominae), commonly known as the bursate nematodes of equines, represent a common and severe threat to the livestock breeding industry worldwide (Lichtenfels et al. 2008; Zhang 2017; Hu et al. 2020). There are 13 valid species in the genus *Cylicocyclus* responsible for such morbidity and socioeconomic burdens. *Cylicocyclus auriculatus* Looss 1900 is a significant member of the *Cylicocyclus* nematodes and frequently found in donkeys (Lichtenfels et al. 2008; Kuzmina and Kuzmin 2008; Bu et al. 2009, 2013). This parasite inhabits in the large intestine of donkeys and can cause abdominalgia, diarrhea, weight loss and even death. Although there have been substantial advances in morphology and biology of *C. auriculatus* so far, knowledge gaps to understand this nematode at the molecular level, especially in its genetics and molecular epidemiology, are still not sufficiently explored because of lacking suitable genetic markers (Lichtenfels et al. 2008; Bu et al. 2013). More importantly, until now the morphology-based classification of *Cylicocyclus* has been controversial (Lichtenfels et al. 2008; Zhang and Kong 2002; Gao et al. 2021). Under this context, we decoded the mitochondrial genome of *C. auriculatus*, a representative member of this genus from the Dezhou donkey in Southwest China using the Illumina sequencing technology, as the mitochondrial DNA is not only a rich resource for molecular markers but its complete data can also provide novel insights into phylogenetic relationships of *Cylicocyclus* (Jex et al. 2010; Gao et al. 2017; Hu et al. 2020; Gao et al. 2021).

A total of six parasite specimens were obtained from a Dezhou donkey housed in an original breeding farm at Dazhou (31°92′N, 103°29′E), Sichuan Province of Southwest China, after treatment with pyrantel pamoate. These specimens were identified as adults of *C. auriculatus* with two males and four females, according to morphological keys of Lichtenfels et al. (2008). One male specimen was used for DNA isolation and the remaining were fixed in 5% formalin solution and archived in the Parasitological Museum of Sichuan Agricultural University (https://dop.sicau.edu.cn/; xyue1985@gmail.com (Yue Xie)) under collection numbers XY2018_28-32. After quality and quantity assessment, ∼2 µg genomic DNA was fragmented to construct a 350-bp paired-end (PE) library, followed by sequencing on an Illumina HiSeq X-TEN platform. The clean reads (∼1.5 Gb) were used to assemble a mitochondrial genome with MITObim (Hahn et al. 2013). Gene annotation was achieved using MITOS (Bernt et al. 2013). The complete genome sequence was deposited in GenBank under accession number: MZ888509.

The mitochondrial genome of *C. auriculatus* was 13,851 bp in size and encoded 12 protein-coding genes (*atp*6, *cox*1-3, *cytb*, *nad*1-6 and *nad*4L), 22 tRNA genes and two rRNA genes (*rrn*L and *rrn*S). All genes were unidirectionally transcribed on the same strand. Among these twelve protein-coding genes, *atp*6, *cytb*, *cox*1-3, *nad*3, *nad*5, *nad*6 and *nad*4L started with ATT, while *nad*1, *nad*2 and *nad*4 used the TTG as the initiation codon. Correspondingly, except for *cox*3 and *nad*2 deduced to end with ‘T’ or ‘TAG’, the remaining genes were predicted to use the TAA as the stop codons. Twenty-two tRNA genes ranged from 54 bp (tRNA^(AGN)^-Ser) to 63 bp (tRNA-Lys) in size. In addition to tRNA-Ser genes, all had a DHU arm and a TV-replacement loop instead of the TψC arm (Xu et al. 2015; Gao et al. 2017, 2021; Li et al. 2019; Qiu et al. 2019; Hu et al. 2020). Two rRNAs, the small rRNA (*rrn*S; 700 bp) and large (*rrn*L; 968 bp) subunits, were located between tRNA-Glu and tRNA^(UCN)^-Ser and between tRNA-His and *nad3*, respectively. Two non-coding regions, namely the long non-coding region (LNCR; 273 bp) and short non-coding region (SNCR; 88 bp), were located between tRNA-Ala and tRNA-Pro and between *nad4* and *cox1*, respectively.

Building on a concatenated amino acid dataset of 12 protein-coding genes from 17 strongyloid parasites, the maximum parsimony (MP), maximum-likelihood (ML) and Bayesian inference (BI) methods were used to construct phylogenetic trees using *Necator americanus* Stiles 1902 as the outgroup because of its relationship with equine Strongyloidea nematodes (Jex et al., 2009; Liu et al., 2013; Gao et al., 2021). The three identical trees clearly grouped *C. auriculatus* with *Cylicocyclus ashworthi* Le Roux 1924, *Cylicocyclus insigne* Boulenger 1917, *Cylicocyclus nassatus* Looss 1900 and *Cylicocyclus radiates* Krecek 2011 and together formed a monophyletic relationship with either species of *Cyathostomum*, *Coronocyclus*, *Cyathostomum*, *Cylicostephanus* or *Cylicodontophorus* within Cyathostominae (Figure 1). Further, *C. auriculatus* was determined to be more closely related to *C. insigne* than to other species in *Cylicocyclus*, with high statistical supports (all bootstrap values ≥99 or =1.00), consistent with recent molecular studies (Hu et al. 2020; Gao et al. 2021), demonstrating the phylogenetic stability of these bursate nematodes found in equines. Taken together, the sequenced mitochondrial genome of *C. auriculatus* not only provides novel molecular evidence for its phylogenetic position in *Cylicocyclus* but also enriches the marker resource for molecular diagnostic, systematic and evolutionary biological studies of strongyloid nematodes.

**Figure 1.**
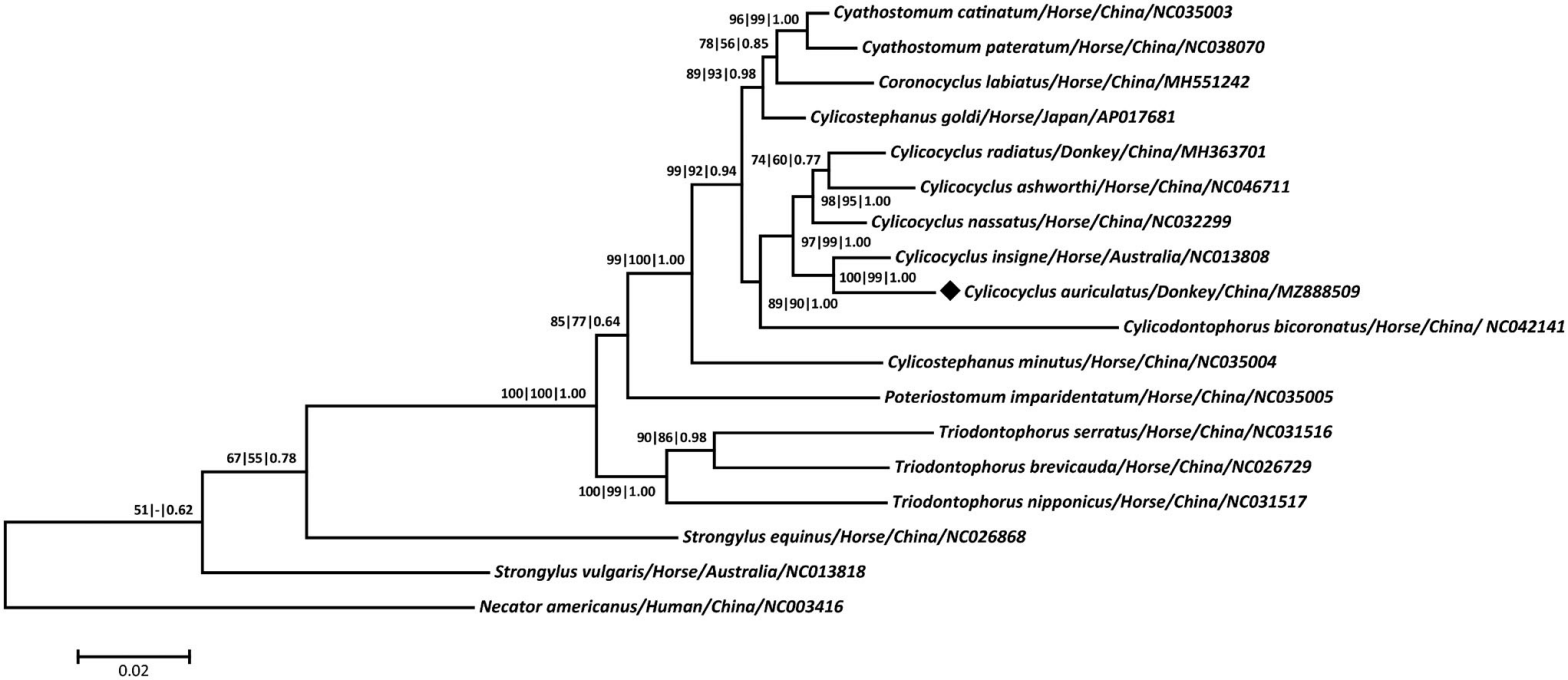
Phylogeny was inferred from maximum parsimony (MP), maximum-likelihood (ML) and Bayesian inference (BI) analyses based on concatenated amino-acid sequences of 12 mt protein-coding genes of *C. auriculatus* and other related nematodes. Numbers along the branches represent bootstrap values calculated from different analyses in the order: MP/ML/BI; values < 50% are not shown. The scale indicates an estimate of substitutions per site, using the optimized model setting. The solid black diamond represents the species in this study.

## Data Availability

The genome sequence data that support the findings of this study are openly available in GenBank of NCBI at https://www.ncbi.nlm.nih.gov, under the accession number MZ888509. The associated BioProject, SRA and Bio-Sample numbers are PRJNA794955, SRR17475793 and SAMN24665689, respectively.
